# A Comprehensive Overview of the Past, Current, and Future Randomized Controlled Trials in Hepatic Encephalopathy

**DOI:** 10.3390/medicina59122143

**Published:** 2023-12-10

**Authors:** Ovidiu-Dumitru Ilie, Raluca Duta, Ilinca-Bianca Nita, Irina Dobrin, Irina-Luciana Gurzu, Irina Girleanu, Laura Huiban, Cristina Muzica, Alin Ciobica, Roxana Popescu, Petru Cianga, Carol Stanciu, Diana Cimpoesu, Anca Trifan

**Affiliations:** 1 Gastroenterology Group, CENEMED Platform for Interdisciplinary Research, University of Medicine and Pharmacy “Grigore T. Popa”, University Street No. 16, 700115 Iasi, Romania; 2 Department of Medicine III, Faculty of Medicine, University of Medicine and Pharmacy “Grigore T. Popa”, University Street No. 16, 700115 Iasi, Romania; 3 Institute of Psychiatry “Socola”, Bucium Street No. 36, 700282 Iasi, Romania; 4 Department of Preventive Medicine and Interdisciplinarity, Faculty of Medicine, University of Medicine and Pharmacy “Grigore T. Popa”, University Street No. 16, 700115 Iasi, Romania; 5 Department of Gastroenterology, Faculty of Medicine, University of Medicine and Pharmacy “Grigore T. Popa”, University Street No. 16, 700115 Iasi, Romania; 6 Institute of Gastroenterology and Hepatology, “St. Spiridon” County Emergency Clinical Hospital, Independence Avenue No. 1, 700111 Iasi, Romania; 7 Department of Biology, Faculty of Biology, “Alexandru Ioan Cuza” University, Carol I Avenue No. 20A, 700505 Iasi, Romania; 8 Centre of Biomedical Research, Romanian Academy, Carol I Avenue No. 8, 700506 Iasi, Romania; 9 Academy of Romanian Scientists, Splaiul Independentei No. 54, Sector 5, 050094 Bucharest, Romania; 10 Preclinical Department, “Apollonia” University, Păcurari Street No. 11, 700511 Iasi, Romania; 11 Department of Medical Genetics, University of Medicine and Pharmacy “Grigore T. Popa”, University Street No. 16, 700115 Iasi, Romania; 12 Department of Medical Genetics, “Saint Mary” Emergency Children’s Hospital, Vasile Lupu Street No. 62, 700309 Iasi, Romania; 13 Department of Immunology, University of Medicine and Pharmacy “Grigore T. Popa”, University Street No. 16, 700115 Iasi, Romania; 14 Department of Emergency Medicine, “St. Spiridon” County Emergency Clinical Hospital, Independence Avenue No. 1, 700111 Iasi, Romania

**Keywords:** hepatic encephalopathy, fecal microbiota transplantation, probiotics, prebiotics, synbiotics, randomized controlled trials

## Abstract

*Background:* Hepatic encephalopathy (HE) caused by cirrhosis has severe consequences on an individual’s lifespan, leading to long-term liver complications and potentially life-threatening outcomes. Despite recent interest in this condition, the effectiveness of secondary prophylaxis involving rixafimin, lactulose, or L-ornithine L-aspartate (LOLA) may be hindered by the unique microbial profiles each patient possesses. *Methods:* Thus, in this manuscript, we aimed to search, identify, and gather all randomized controlled trials (RCTs) published between 2000–2023 (November) in four major academic databases such as PubMed, ISI Web of Science, Scopus, and ScienceDirect by using a controlled terminology and web strings that reunite six main keywords. We complementarily retrieved data on the ongoing RCTs. *Results:* Regardless of the relatively high number of results displayed (*n* = 75), 46.66% (*n* = 35) were initially deemed eligible after the first evaluation phase after removing duplicates, *n* = 40 (53.34%). At the second assessment stage, we eliminated 11.42% (*n* = 4) studies, of which *n* = 22 finally met the eligibility criteria to be included in the main body of the manuscript. In terms of RCTs, otherwise found in distinct stages of development, *n* = 3 target FMT and *n* = 1 probiotics. *Conclusions:* Although we benefit from the necessary information and technology to design novel strategies for microbiota, only probiotics and synbiotics have been extensively studied in the last decade compared to FMT.

## 1. Introduction

HE describes an advanced chronic liver disease (ACLD)-derived syndrome that disrupts the central nervous system (CNS) network, characterizing a life-threatening but reversible heterogenous spectrum of nonspecific features that stem from liver damage. HE-affected individuals exhibit specific clinical signs that reflect on phenotype severity and range from lack of actual manifestations with subtle psychometric scores to neuropsychiatric features involving cognitive decline and motor impairments, leading to coma and death [1,2,3].

The American Association for the Study of Liver Diseases (AASLD) and the European Association for the Study of the Liver (EASL) revised HE into three categories: type A—acute liver failure (ALF), type B—portosystemic bypass or shunt, and type C—portal hypertension and cirrhosis [3,4,5].

Current classification criteria and nomenclature delineate distinctive guiding prospects regarding HE’s stage: minimal HE (MHE) and covert HE (CHE)—grade I—and overt HE (OHE)—grades II-IV—based on the West Haven Criteria. Therefore, HE may lead, in a time-dependent perspective, to an episodic form (1 episode/6 months) with no identifiable precipitants or be triggered by combined effects of subclinical changes, manifesting as recurrent (≥2 episodes/6 months), or persistent, as the most aggressive form [3,6,7,8].

The latest statistics indicate an occurrence that oscillates from 30% to 45% in cirrhotic patients, with 5–25% in the first five years, 7–42% within one decade, and 10% to 50% among individuals who underwent transjugular intrahepatic portosystemic shunt (TIPS). The overall prevalence of C/MHE is 40.9% [9], 10% to 14% in the general population, with 16–21% and 10–50% in decompensated and TIPS patients [3]. However, in other studies, the ratios fluctuate from 20 to85%, and OHE between 30 and40% [3,10,11,12].

Women display higher rates of HE than men (11.5% [13] to 17.8% [14] in contrast with 10.1–16.8%), which further explains the likeliness to not undergo liver transplantation (LT) [15] and lower self-rated health than their counterparts [16].The survival rate projections at 1- and 3-year follow-up vary from 42% to 23%, with OHE recurrence of 42% in the first year and with a trend of mortality within the same interval. It is noteworthy that the number and severity of HE episodes reach about 50% [3,6,12,17].

HE ranks as the third most debilitating condition in decompensated cirrhotics, causing poor health-related quality of life and an increase in the risk of hospitalization and death [2,6]. There is a significant influence on daily routine activities besides the economic burden and pressure on health systems and caregivers regardless of the standard of care (SOC) therapy [18].

Though current management strategies involve SOC treatment, particularly lactulose and rifaximin, no definitive consensus has been accepted to control secondary prophylaxis in routine practice. Rifaximin and lactulose are practical in controlling acute, chronic and recurrent portosystemic HE and MHE [17,19,20,21,22] with data indicating that both administered in parallel are the best-documented option to maintain remission [3]. However, the benefits are counterbalanced by production costs, safety concerns, and side effects [23,24].

The pathophysiology of HE remains challenging to maintain, owing to its development and onset despite SOC [25,26]. It has been recently suggested that HE may arise from alterations of the gut-brain axis (GBA) due to environmental weakening [27,28] and failure of liver detoxification [29]. Complementary investigations led to findings that pinpoint main contributors that include systemic inflammation, endotoxemia, and gut-synthesized end-product breakdown accumulation following protein digestion, amino acid deamination, and bacterial urease activity [10]. These processes ensure glutamine (Gln) uptake in the intestine and the action of Gln secondary to ammonia (NH3) and glutamate (Glu) [30].

Considering the fulminant ascension in the current literature of the GBA link and interconnection with the liver, this particular approach offered accessibility to modulate the microbial milieu, paving the way for intestinal-targeted protocols. The most powerful tools developed to shape the microbial landscape include fecal microbiota transplantation (FMT) and the administration of exogenous supplements such as pro-, pre-, and synbiotics. Thus, the present manuscript aims to offer an up-to-date overview of all clinical trials, particularly randomized controlled trials (RCTs), with the main objective of assessing their reliability in HE.

## 2. Methodology

This manuscript has been conducted following the work of Green et al. [31] on writing a narrative review.

### 2.1. Vocabulary, Academic Databases, and Searching Strategy

To enhance the coverage process in identifying all relevant studies published between 2000 and 2023, we conducted a thorough search in four central academic databases—PubMed, ISI Web of Science, Scopus, and ScienceDirect.

Subsequently, we accessed https://classic.clinicaltrials.gov/ct2/home (accessed on 10 November 2023) as the main source dedicated to reporting privately and publicly funded studies around the world. In this case, we used the following parameters for each field domain: Condition or disease “Hepatic Encephalopathy”, Other terms “Fecal Microbiota Transplantation, Probiotics, Prebiotics, Synbiotics”, Status “Not yet recruiting, Recruiting, Enrolling by invitation, Active, not recruiting”, Study Type “Interventional”, Study Phase “Early Phase I, Phase I, Phase II, Phase III, Phase IV”.

To improve the collection of a significant body literature, we applied a controlled vocabulary and scientific terminology of dedicated keywords. Specifically, each string for analysis was carried out containing “Hepatic Encephalopathy” concomitant with “Fecal Microbiota Transplantation”, “Probiotics”, “Prebiotics”, and Synbiotics” and followed by “Randomized Controlled Trial (RCTs)”, excluding PubMed for which the option of selecting RCTs is included.

The adopted PubMed string was: Hepatic Encephalopathy [Title/Abstract] AND Fecal Microbiota Transplantation [Title/Abstract] OR Probiotics [Title/Abstract] OR prebiotics [Title/Abstract] OR synbiotics [Title/Abstract] AND Randomized Controlled Trials [Title/Abstract]. The adopted ISI Web of Science, Scopus, and ScienceDirect strings were: Hepatic AND Encephalopathy [Title/Abstract] AND Probiotics [Title/Abstract] OR Prebiotics [Title/Abstract] OR Synbiotics [Title/Abstract] AND Randomized AND Controlled AND Trial [Title/Abstract].

### 2.2. Inclusion and Exclusion Criteria

This manuscript included only RCTs published between 2000–2023 (November) written exclusively in English. Other types of research or literature syntheses (quantitative or qualitative) or in another language were automatically excluded.

### 2.3. Number of Entries

**Figure 1:**
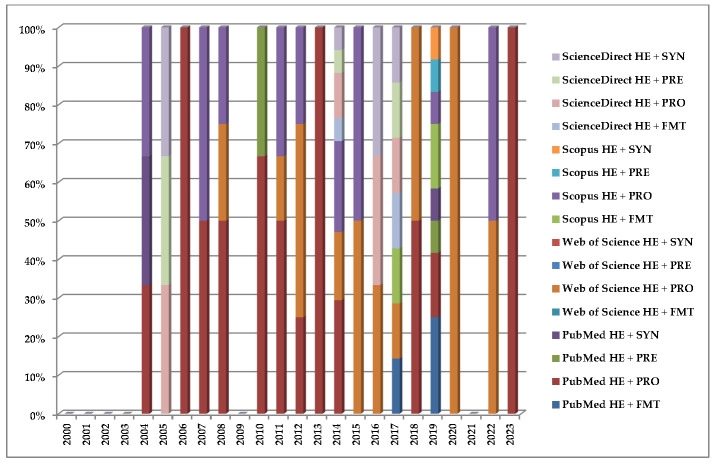
Stacked columns diagram with the published studies between 2000–2023 based on the database and combination of keywords.

Following the assessment and centralization of the returned entries, seventy-five articles were considered eligible for inclusion (Figure 1), from which *n* = 30 (40%) were published in PubMed, *n* = 13 (17.33%) in ISI Web of Science, *n* = 18 (24%) in Scopus, and *n* = 14 (18.66%) in ScienceDirect.

**Figure 2:**
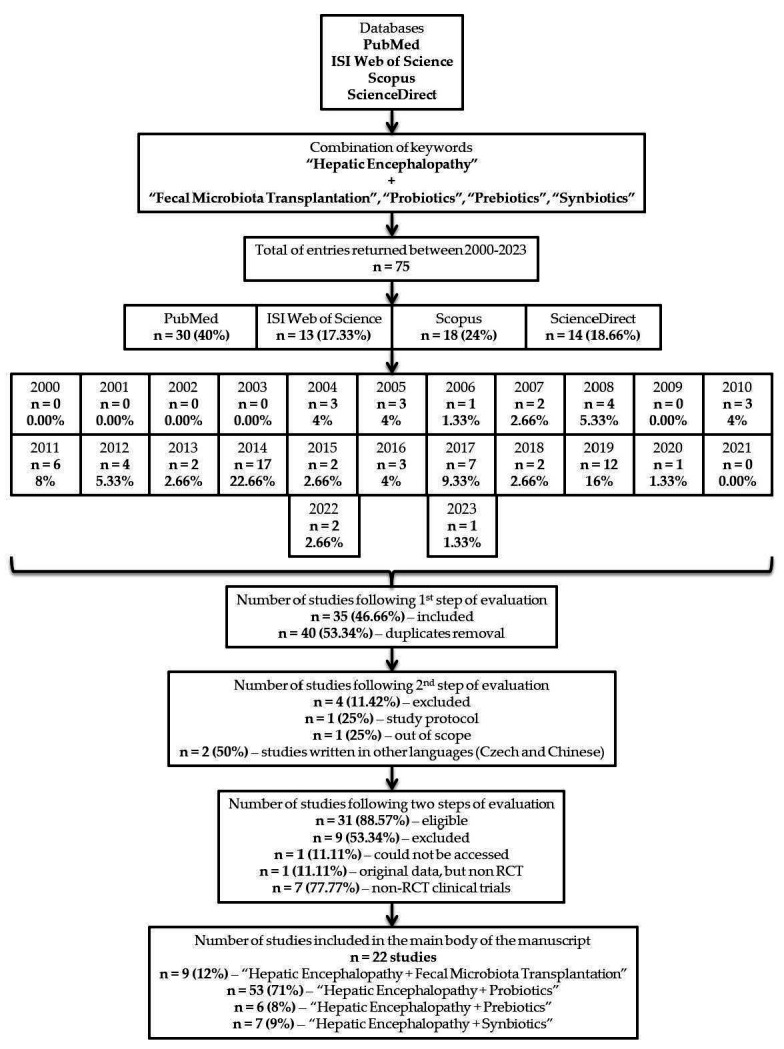
A flowchart highlighting the overall structure of this manuscript.

Taken in a retrospective manner to observe the trend evolution of how many articles have been published in the pre-defined interval, we had the following situation per year: *n* = 0 from 2000 to 2003 (0.00%), *n* = 3 (4%)—2004, *n* = 3 (4%)—2005, *n* = 1 (1.33%)—2006, *n* = 2 (2.66%)—2007, *n* = 4 (5.33%)—2008, *n* = 0 (0.00%)—2009, *n* = 3 (4%)—2010, *n* = 6 (8%)—2011, *n* = 4 (5.33%)—2012, *n* = 2 (2.66%)—2013, *n* = 17 (22.66%)—2014, *n* = 2 (2.66%)—2015, *n* = 3 (4%)—2016, *n* = 7 (9.33%)—2017, *n* = 2 (2.66%)—2018, *n* = 12 (16%)—2019, *n* = 1 (1.33%)—2020, *n* = 0 (0.00%)—2021, *n* = 2 (2.66%)—2022, and *n* = 1 (1.33%)—2023. However, only thirty-five studies (46.66%) advanced to the next phase of evaluation after removing *n* = 40 (53.34%) duplicates. At the second step, *n* = 4 were subsequently removed (11.42%): *n* = 1 (25%)—study protocol, *n* = 1 (25%)—out of scope, and *n* = 2 (50%)—studies written in other languages (Czech and Chinese). Depending on the intervention the patients were subjected to, we identified *n* = 9 (12%)—“HE + FMT”, *n* = 53 (71%)—“HE + PROBIOTICS”, *n* = 6 (8%)—“HE + PREBIOTICS”, and *n* = 7 (9%)—“HE + SYNBIOTICS (Figure 2).

### 2.4. Number of Results

After two phases of evaluating the studies that preliminarily met the inclusion criteria to be added to the main body of the manuscript, only *n* = 31 (88.57) were further considered. Though we completed the fields for excluding any other type of manuscript, except RCTs, multiple studies were additionally excluded; *n* = 1 (11.11%)—could not be accessed, *n* = 1 (11.11%)—was an original, but non-RCT, while *n* = 7 (77.77%)—were clinical trials, but divergent with the primary directions of our manuscript (Figure 2). An overview of all the studies is presented in Table 1. Also, a list of all reported HE precipitants can be consulted in Table 2.

## 3. Results and Discussions

### 3.1. FMT

Bajaj and collaborators previously focused on the potency of FMT in modulating the microenvironment, based on a series of primary objectives and endpoints. The primary focus was oriented on evaluating the safety and tolerability of FMT while also exploring its influence on brain function and the mucosal/stool microbiota [34] with particular emphasis on bile acids (BAs), inflammation, and association with EncephalApp [35]. The success rate using a rationally derived stool donor [32] and the long-term impact on cognition and number of hospitalizations [33] have been a priority. Thus, enrolled participants were organized to receive either fifteen FMT capsules or 90mL frozen-then-thawed units. As anticipated, the effects following the intervention in recipients arose in a relatively brief timeframe compared with the placebo/SOC groups.

However, sixteen patients from both subsets required further medical attention after the study’s completion. In light of the outcomes, the FMT capsules led to an increase in the diversity of *Ruminococcaceae* and *Bifidobacteriaceae* in the duodenal mucosa, alongside a decrease in the ratio of *Streptococcaceae* and *Veillonellaceae*, apart from *Veillonellaceae* in the sigmoid and stool, as the proportion was below average. Representatives of the families *Ruminococcaceae*, *Verrucomicrobiaceae*, and *Lachnospiraceae* were linked to cognitive function and EncephalApp performance, by down-regulating interleukin-6 (IL-6) and serum endotoxins such as lipopolysaccharide (LPS)-binding protein (LBP), unlike duodenal E-cadherin and defensin alpha 5 (DEFA5) [34,35].

Research in the field advanced the concept of the so-called keystone species that rely upon a longer follow-up. This strategy enclosed a pre-treatment interlude of five days with broad-spectrum antibiotics that consisted of lactulose, rifaximin, and proton pump inhibitors (PPIs). The protocol had to be discontinued for seven participants. Ten experienced severe adverse effects (SAEs), and two were independent from the FMT since the enema compensated for the decline of taxa and diversity with *Proteobacteria* dominance. Conversely, the SOC group had not experienced significant improvements in cognition or Model for End-Stage Liver Disease (MELD) score [32]. Subsequent analyses confirmed the potential of this technique, as there had been an elevation in the relative abundance of *Burkholderiaceae* and similar *Lachnospiraceae* and *Ruminococcaceae* with a decline of *Acidaminococcaceae.* Microbial shifts prevented the risk of recurrence and enhanced cognitive functioning, as displayed by EncephalApp, lessening hospitalization requirements of those who accepted FMT [33].

Microbial conversion of Bas might be beneficial in surveying the outcomes owing to the high deconjugation and 7α-dehydroxylation by FMT stimulation not seen in decompensated cirrhotic patients [54] as a result of a relative reduction in *Clostridial* spp. [54,55]. It is important to decipher, in the current context, the alterations that correlate with pathogenesis and prognostication in HE [3,56] and immunoinflammatory mechanisms [57]. *Akkermansia muciniphila* is responsible for enhancing the intestinal barrier and inflammatory responses in patients with or without liver disease [58,59,60,61]. However, there are subsets of patients that experienced progressive cognitive degradation even after SOC [62] and had odds of it being irreversible [63] following LT, as well as the possibility of readmission, which should be appropriately treated [64,65].

Microorganism species originating in the oral cavity such as *Streptococcaceae* and *Veillonellaceae* are often found in the intestinal mucosa and stool of cirrhotic patients [66,67,68,69] and are usually associated with poor prognosis and exacerbation by the PPIs [67,68]. *Veillonella* spp. can contribute to the pathogenesis of gram-negative bacilli, which may result in a lack of rifaximin efficiency and PPI withdrawal [70,71,72], with *Streptococcaceae* expressing urease that potentially generates ammonium (NH_4_) [71,73]. On the other hand, *Lachnospiraceae*, *Ruminococcaceae*, and *Bifidobacteriaceae* families under normal eubiosis state partake in short-chain fatty acids (SCFAs) synthesis and BA metabolism [74,75].

Infections and antibiotics are the main precursors of liver injury, illustrated by the levels of urinary phenylacetylglutamine (PAG), hippurate, and formate. The latter two are associated with *Proteobacteria* and *Firmicutes*, respectively, and the bacterial degradation of phenylalanine [76]. However, additional studies are necessary, relying on a strict screening assessment that must meet the requirements of Openbiome [77,78].

### 3.2. Synbiotics

Liu et al. [36] conducted a study on the quantitative bacteriological ecology dysregulation in contrast with conventional culture-based methods. Synbiotics are a class of supplements that reunite pro- and prebiotics—fermented fibers that enrich the proliferation of non-urease-producing *Lactobacillus* since they restrain the NH3-producing microorganism’s effects and prevent endotoxemia. Briefly, they confine fecal overgrowth of pathogenic *Escherichia coli* and *Staphylococcus* spp. Depending on the colonic pH reduction, synbiotics hinder the progression of liver injuries and decrease serum NH3 levels as an alternative to reverse MHE. *Lactobacillus paracasei*, *Lactobacillus plantarum*, *Leuconostoc mesenteroides*, and *Pediococcus pentosaceus,* in combination with branched-chain amino acids (BCAAs), improve the performance on psychometric tests such as the Trail Making Test (TMT) and Inhibitory Control Test (ICT) as indicated by scores indicate in an outpatient setting for patients with OHE, as suggested by Vidot et al. [37].

The development of multiresistant microorganisms, as a consequence of the long-term use of non-absorbable antibiotics and the approach to counter this consequence, may involve overlapping episodes of rifaximin and off-treatment periods with probiotics according to one of the earliest studies by Lighthouse et al. [79]. There is a scarce body of literature on the topic regarding the evaluation of synbiotics on nutritional parameters after LT. Intriguingly, only three original studies by Rayes [80,81] and Zhang et al. [82] argued an incidence reduction of post-operative bacterial wound infections and briefer antibiotic therapy. Even sole supplementation with probiotics from enlistment until transplantation has been verified to be a reliable option, as early improvements on biochemical parameters were observed. A lower bilirubin concentration early postoperatively promotes a faster allograft function, as evidenced by a decrease in plasma alanine aminotransferase (ALT) and aspartate (AST) per Grąt et al. [83].

Bacterial infections are among the most common causes of morbidity in the first three months of post-operative LT. Age as a self-standing parameter or in combination with liver or renal diseases, malnutrition, or a high number of transfusable blood products also exerts a significant influence on the overall risk. A rather customary practice that involves *Lactobacillus plantarum* 299 and oat fiber might be helpful in surgical patients to prevent pancreas necrosis or abscess [84].

A subject that generated debate and contradictions was the length of probiotic administration to attain an optimal outcome, as that several reports claimed the emergence of the first signs after a few days [85,86]. Contrariwise, others speculated the advance of valid preventive effects after regular consumption [87,88] or longer intervals in cirrhotic patients [36]. From the above considerations, probiotics were classified as the most potent tool from which cirrhotic individuals benefit, especially those post-LT. A combination of multiple lactic acid bacteria strains minimized the chances of infection before and after surgery [89,90,91], findings that aligned with previous research, but with no significant protective impact against *Clostridium difficile* [83], as another group emphasized [92].

The potential of probiotics during the pre-transplant interval was a topic already discussed, as well as their influence on the expression and levels of various biochemical markers. Separated from bilirubin concentration normalization, they impact transaminase activity and relieve graft injury in rats. Though data concerning the developments of perioperative administration were lacking, the favorable imprint of probiotics during pre-operative settings was well verified [80,81,93]. It should be recognized that results were only partially in agreement with previous evidence on biochemical levels in patients with non-alcoholic fatty liver disease (NAFLD) or alcohol-induced liver injury involving improved regeneration after liver resection [94,95,96,97,98,99]. In other words, the effectiveness of a treatment depends on the constitutive strains. A *Saccharomyces boulardii* regime for 30 days did not lead to the anticipated outcomes in patients eligible for TL [100].

### 3.3. Probiotics

Bajaj et al. [40,45] supported the concept of a controlled *Lactobacillus* spp. intake to enhance the host’s health status. Yogurt consumption reduced the episode numbers, with no notable adverse effects (AEs), and successfully reversed the MHE. This was verified by the scores in the Psychometric Test-A (NCT-A), Block Design Test (BDT), and Digit Symbol Test (DST), as opposed to the group that developed OHE [40]. *Lactobacillus* GG is a potent strain in re-establishing the host’s eubiosis since it decreases *Enterobacteriaceae* and correlates with the increase of *Lachnospiraceae* and Clostridiales Incertae Sedis XIV. Besides the noteworthy modifications in the stool microbial profile, it further enables a decline of endotoxemia, and attenuates cytokine level of tumor necrosis factor α (TNF-α) without improving cognitive capacity in cirrhotic MHE patients [45].

Analogous to the efforts in alleviating the severity of this phenotype, Malaguarnera et al. [38,41] tested the possible benefits of *Bifidobacterium longum* plus fructooligosaccharides (FOSs) in parallel to the secondary prophylaxis lactulose. Eligible individuals exhibited modifications not seen in prophylactic settings, notably a decrease in the Trail Making Test B (TMT B) (1 month), NH4 fasting HE1, Trail Making Test A (TMT A), TMT B (2 months), and an increase in the Symbol Digit Modalities Test (SDMT) and Block Design Test (1–2 months).

A publication that targeted decompensated hepatitis B-induced cirrhotic patients without OHE revealed that indirect methods of elevating the amount of SCFAs beneficial bacteria may be valuable. Xia et al. [50] highlighted that *Clostridium butyricum* (CGMCC0313-1) combined with *Bifidobacterium infantis* (CGMCC0313-2) supports the intestinal barrier, thus preventing permeability as D-lactate, LPS, and diamine oxidase (DAO) levels indicate. This explains the reduction in venous NH3 and scores of NCT-A and DST in MHE patients, considering the shifts between the pathogenic *Enterococcus* and *Enterobacteriaceae* towards *Clostridium* cluster I and *Bifidobacterium*.

VSL#3 is a central probiotic that reunites a mixture of *Lactobacillus* spp. (*paracasei* DSM 24733, *plantarum* DSM 24730, *acidophilus* DSM 24735, and *delbrueckii* subspecies bulgaricus DSM 24734), *Bifidobacterium* (*longum* DSM 24736, *infantis* DSM 24737, and *breve* DSM 24732) and *Streptococcus* spp. (*thermophilus* DSM 24731) genera. An appropriate dosing shortens hospital visits in patients with cirrhosis and HE by improving Child-Pugh-Turcotte (CTP), Psychometric HE (PHES), and MELD scores. In addition, VSL#3 prevents bacterial translocations and pro-inflammatory adhesion of molecules and cytokines, as suggested by plasma levels of IL-1β, IL-6, TNF-α, aldosterone, renin, brain natriuretic peptide (BNP), NH_3_, and indole, as Dhiman et al. [48] demonstrated. Before the observations of Dhiman and his colleagues, Lunia et al. [46] assigned patients who had not experienced HE to undergo probiotic VSL#3 or to go without intervention, and intriguingly, individuals from both subsets developed OHE. Apart from the improvement in psychometric scores, this combination exerted numerous beneficial effects among which were the reduced levels of arterial NH_3_, ameliorated small intestinal bacterial overgrowth (SIBO), shortened orocecal transit time (OCTT), and number needed to treat (NTT). Pratap Mouli et al. [49] performed several analyses and concluded that VSL#3 is non-inferior compared to the standard therapeutic lactulose, as serum NH_3_ between the groups shows an antithetical relationship. The efficacy of the same product has been subsequently inquired by Román et al. [51]. A controlled regime monitored gait speed, cognitive functions, Timed Up and Go (TUG), and PHES scales, with also a notable down-regulation of pro-inflammatory cytokines. This included C-reactive protein (CRP), TNF-α, intestinal permeability markers such as fatty acid binding protein 6 (FABP-6), and claudin-3, completed by an unexpected increase in neutrophil oxidation.

Recent reports from the same unit accentuated the differential mark of probiotics alone or together with lactulose or no therapy [39,44] and by comparison with LOLA [43] and rifaximin [47]. Sharma et al. [39,101] expressed that a combination of probiotics with or without lactulose is equally practical in relieving symptoms of MHE as indicated by the psychometric scores, normalization of P300 auditory event-related potential (P300ERP), and venous NH3. Mittal et al. [43] substantiated the findings and underlined that LOLA reverses MHE and improves health-related quality of life (HRQOL), Sickness Impact Profile (SIP) questionnaire, and reduces arterial NH3 levels. Previous results also pinpointed the role of this synbiotic as the recurrence rate was similar among those who underwent treatment and recovered from an episode of OHE and it prevented additional evolution, as stated by Agrawal et al. [44]. Sharma et al. [47] confirmed all the conclusions as they proved an improvement in the psychometric tests and Critical Flicker Frequency (CFF) scores, which imply the possibility of including probiotics as a secondary prophylactic alternative.

Difficulties with palatability and compliance [102,103] followed by bloating, flatulence, cramping, abdominal pain, diarrhea, and nausea [103,104] are among the prominent forerunners for short-term adherence (80%) [105]. Gut flora metabolism-derived nitrogenous products such as NH3, mercaptans, endotoxins, or benzodiazepine-like compounds, notably blood NH_3_ in the bowel, result from degradation by aerobic and anaerobic bacteria. These products are directed to the liver through portal flux and are eliminated as urea.

Another mixture of strains that reunites *Lactobacillus acidophilus*, *Lactobacillus rhamnosus*, *Bifidobacterium longum*, and *Saccharomyces boulardii* did not elicit pertinent advancements in any of the parameters of interest in MHE patients; no considerable dissimilarities in the arterial NH3 evoked responses and number connection tests. The data should be interpreted with caution, since Saji et al. [42] did not define the MHE respecting the consensus guidelines. Given the gap in evidence regarding specific strains, it is challenging to generalize recommendations. The probiotic fungus *Saccharomyces boulardii*’s impact on inflammatory biomarkers was evaluated and appears to stimulate a decrease in serum CRP and ameliorates hyperdynamic circulation in decompensated cirrhotic patients. The relative abundance of *Coprococcus*, *Senegalimassilia*, and *Desulfovibrio* lowered, with the mention that the latter is responsible for body mass index (BMI), waist size, triglyceride, and uric acid levels, per Maslennikov et al. [53]. In addition, *Lactobacillus casei* Shirota-treated patients exhibited a marked reduced plasma monocyte chemotactic protein-1 (MCP-1), IL-1β, IL-17a, and macrophage inflammatory protein-1β (MIP-1β), as indicated by Macnaughtan et al. [52].

Pereg et al. [106] affirmed that compensated cirrhotics may not directly benefit from probiotics supplementation, which was substantiated by clinical and laboratory tests, but rather may benefit from a slight trend of reduction in serum NH3 above the normal baseline. Maharishi et al. [107] conceived a design in an outpatient setting aiming to enroll MHE patients who were advised to follow a diet with a caloric restriction in the framework of nutritional therapy through constant dietician visits and guidance-based educational materials. The vegetable and casein-based diet regulated the frequency of OHE episodes, thus reflecting in the HRQOL and hospitalization number via the contribution of related mechanisms in contrast with nutritional therapy in healthy individuals. LOLA bears its utility as a prophylactic in post-transjugular intrahepatic portosystemic stent shunt (TIPSS), having a potent effect against HE recurrence in patients experiencing episodes of OHE with longer duration until the first breakthrough, as Varakanahalli et al. [108] discovered.

It is important to acknowledge that avoiding protein may not be the adequate resolution for treating HE since it could negatively affect the energy metabolism, especially in cases of prolonged nitrogen retention [109]. There were no considerable discrepancies between patients with cirrhosis that underwent nutritional support and those without intervention [110,111]. An approach to improve cognition, executive functions, energy metabolism, nutritional parameters, and HRQOL in MHE patients may reside in adopting personalized dietary habits, such as breakfast with the necessary amount of protein, long-term late-evening snacks, and frequent meals [112,113,114,115].

## 4. Current Status of RCTs

Four RCTs are currently underway in different stages of completion, of which *n* = 3 target FMT (ClinicalTrials.gov Identifier: NCT03420482, ClinicalTrials.gov Identifier: NCT03796598, and ClinicalTrials.gov Identifier: NCT04932577) and *n* = 1 probiotics (ClinicalTrials.gov Identifier: NCT05539027). Among these RCTs, a total of *n* = 450 individuals are expected to register, having as implementation points two centers from the United States (Massachusetts General Hospital Boston, Massachusetts/Hunter Holmes McGuire VA Medical Center, Richmond, Virginia), Denmark (Department of Hepatology and Gastroenterology, Aarhus University Hospital Aarhus) and Egypt (Ain-Shams University Hospitals Cairo). NCT03420482 found in Phase II has an estimated completion date of 31 January 2024, and includes *n* = 30 individuals, NCT03796598 in Phase I and Phase II to be completed on 31 December 2024 with *n* = 60 individuals, while NCT04932577 in Phase II and Phase III to be completed on 31 May 2027 with *n* = 220 individuals. On the other hand, NCT05539027 is already in Phase IV and has an estimated completion date of 30 November 2023, and includes *n* = 140 individuals.

## 5. Conclusions

The associated phenotype exhibited by patients with cirrhosis following HE development may be alleviated via the intimate networks between the brain and gut/liver axes. Whether administered exclusively orally or involving more complex working protocols with frozen or encapsulated human matter in a lyophilized state, it is indicated these approaches receive high priority in clinical practice as they possess numerous advantages, including mild-to-absent complications, safety, and well-toleration by the recipients. FMT via enema retains the microbial balance, reverses the pro-inflammatory landscape, and boosts cognitive functioning compared with SOC due to the low risk of recurrence and SAE episodes. Synbiotics fulfill pivotal roles in regulating internal microbial communities and alleviate the risks of possible bacterial overgrowth and/or endotoxemia. Probiotics, especially *Lactobacillus* and *Bifidobacterium* demonstrated on multiple occasions to be non-inferior in contrast with actual management drugs. These alternative therapeutic applications particularly improve an individual’s state of health as reflected by the levels of pro-inflammatory biomarkers, biochemical parameters, psychometric tests, and microorganism ratios. Although there is a gap in our knowledge, this topic offers opportunities to develop dedicated management strategies. Emerging treatment techniques may crystallize in the patient’s quality of life (QOL). Future RCTs hopefully will pave the way towards novel research and clinical perspectives.

## Figures and Tables

**Table 1:** A synthesized presentation of the eligible studies according to the registration number in https://clinicaltrials.gov/, total number of patients, randomization, intervention, donor, and follow-up in chronological order.

Registered Number	Total Number of Patients	Randomization/ Allocation	Intervention	Donor	Follow-Up	Reference
**FMT**						
NCT02636647	*n* = 80 patients *n* = 20 per study	1:1	frozen-then-thawed FMT	A single donor enriched in *Lachnospiraceae* and *Ruminococcaceae*	5 months	[32]
NS					>12–15 months	[33]
NCT03152188			capsular FMT		5 months	[34]
					5 months	[35]
**SYNBIOTICS**						
No	*n* = 55 patients	*n* = 20 Synbiotics *n* = 20 Fermentable fiber *n* = 15 Placebo	30 days of *Pediococcus pentosaceus, Leuconostoc mesenteroides*, *Lactobacillus paracasei* subspecies *paracasei*, and *Lactobacillus plantarum* of 10^10^ CFU + 10 g of fermentable fiber (2.5 g per beta glucan, inulin, pectin, and resistant starch) each		NS	[36]
ACTRN12610001021066	*n* = 61 initially included, but remained *n* = 49 patients	*n* = 12 Synbiotics *n* = 12 BCAAs *n* = 13 Synbiotics + BCAAs *n* = 12 Placebo	*Pediococcus pentosaceus*, *Leuconostoc mesenteroides*, *Lactobacillus paracasei* subspecies *paracasei*, and *Lactobacillus plantarum* of 10^11^ CFU + 10 g of fermentable fiber (2.5 g oat bran, pectin, inulin, and resistant starch) each 10 g crystalline starch for placebo		At 1–2 months	[37]
**PROBIOTICS**						
No	*n* = 66 initially included, but remained *n* = 60 patients	*n* = 30 *Bifidobacterium longum* + FOS *n* = 30 Placebo	30–60–90 days of *Bifidobacterium longum* W11 + FOS (2.5 g), and vitamin B1 (1.4 mg), vitamin B2 (1.6 mg), vitamin B6 (2.0 mg), vitamin B12, (1.0 mg)		After 30 days	[38]
No	*n* = 240 patients	*n* = 50 Controls *n* = 190 Cirrhotics initially included, but remained *n* = 105	1 month of 30–60 mL lactulose/day; 1 capsule 3 times/day *Streptococcus faecalis* 6 × 10^7^, *Clostridium butyricum* 4 × 10^6^, *Bacillus mesentricus* 2 × 10^6^, lactic acid bacillus 1 × 10^8^ 30–60 mL lactulose + *Streptococcus faecalis* 6 × 10^7^, *Clostridium butyricum* 4 × 10^6^, *Bacillus mesentricus* 2 × 10^6^, lactic acid bacillus 1 × 10^8^;		1 month	[39]
No	*n* = 66 initially included, but remained *n* = 25	*n* = 8 No treatment *n* = 17 Yogurt	60 days of *Streptococcus thermophilus*, *Lactobacillus bulgaricus*, *Lactobacillus acidophilus*, *Lactobacillus casei* *Bifidobacteria*		Initial, and after 30–60 days	[40]
No	*n* = 125 patients	*n* = 62 patients Lactulose *n* = 63 *Bifidobacterium* + FOS	30–60 days of *Bifidobacterium* + FOS lactulose		After 30–60 days	[41]
No	*n* = 43 patients	*n* = 21 Probiotics *n* = 22 Placebo	3 times/day for 4 weeks of *Lactobacillus* *acidophilus*, *Lactobacillus rhamnosus*, *Bifidobacterium longum*, *Sacharomyces boulardi* 1.25 × 10^9^		NS	[42]
No	*n* = 422 initially included, but remained *n* = 322	*n* = 162 No MHE *n* = 160 MHE (*n* = 40 No treatment, *n* = 40 Lactulose, *n* = 40 Probiotics, *n* = 40 LOLA)	3 months of standard treatment divided in dose for stool frequency (2–3 semisolid stools) 30–60 mL lactulose probiotics 1.1 × 10^11^ CFU 6 g LOLA 3 times/day		After 3 months	[43]
No	*n* = 360 initially included, but remained *n* = 235	*n* = 77 Probiotics *n* = 78 No therapy *n* = 80 Lactulose	30–60 mL lactulose (2/3 doses to pass 2–3 semisoft stools) three capsules/day 1.125 × 10 of *Lactobacillus casei*, *Lactobacillus plantarum*, *Lactobacillus acidophilus*, *Lactobacillus delbrueckii* subsp. *bulgaricus*, *Bifidobacterium longum*, *Bifidobacterium breve*, *Bifidobacterium infantis*, *Streptococcus salivarius* subsp. *thermophiles* no therapy		After 3 months/12 months	[44]
No	*n* = 150 initially included, but remained *n* = 30	ITT *n* = 18 LGG *n* = 19 Placebo Per protocol *n* = 14 LGG *n* = 16 Placebo	*Lactobacillus* GG > 5 × 10^10^ CFU/g (5.1 × 10^10^, 5.3 × 10^10^, 6.1 × 10^10^)		Initial, and at 4–8 weeks	[45]
CTRI/2012/07/002807	*n* = 160 patients	*n* = 74 Control *n* = 86 Probiotics	1 capsule 3 times/day of *Bifidobacterium breve*, *Bifidobacteriumlongum*, *Bifidobacterium infantis*, *Lactobacillus acidophilus*, *Lactobacillus plantarum*, *Lactobacillus paracasei*, *Lactobacillus bulgaricus*, *Streptococcus thermophilus* 1.1 × 10^11^ CFU		After 3–6 months	[46]
No	*n* = 317 initially included, but remained *n* = 124	*n* = 30 Placebo *n* = 31 LOLA *n* = 31 Rifaximin *n* = 32 Probiotics	2 months of 18 g LOLA 2 sachets 3 times/day 400 mg rifaximin 3 times/day 5.0 × 10^9^ CFU of *Lactobacillus acidophilus* (0.7 × 10^9^), *Lactobacillus rhamnosus* (0.6 × 10^9^), *Lactobacillus plantarum* (0.6 × 10^9^), *Lactobacillus casei* (0.6 × 10^9^), *Bifidobacterium longum* (0.6 × 10^9^), *Bifidobacterium infantis* (0.6 × 10^9^), *Bifidobacterium breve* (0.6 × 10^9^), *Streptococcus thermophilus* (0.6 × 10^9^), *Sacchromyces boulardi* (0.1 × 10^9^)		For 2 months	[47]
No	*n* = 221 initially included, but remained *n* = 130	*n* = 64 Placebo *n* = 66 Probiotics	1 month of *Lactobacillus paracasei* DSM 24733, *Lactobacillus plantarum* DSM 24730, *Lactobacillus acidophilus* DSM 24735, *Lactobacillus delbrueckii* subspecies *bulgaricus* DSM 24734, *Bifidobacterium longum* DSM 24736, *Bifidobacterium infantis* DSM 24737, *Bifidobacterium breve* DSM 24732, *Streptococcus thermophilus* DSM 24731 of 9 × 10^11^ CFU 24 weeks ofcorn flour placebo		For 6 months	[48]
NCT01008293	*n* = 562 initially included, but remained *n* = 227	*n* = 107 Without MHE *n* = 120 MHE (*n* = 60 Lactulose *n* = 60 Probiotics)	2 months of 30–60 mL/day (2/3 soft stools) 2 capsules/day of 4.5 × 10^11^ CFU of *Lactobacillus acidophilus* DSM 24735, *Lactobacillus plantarum* DSM 24730, *Lactobacillus paracasei* DSM 24733, *Lactobacillus delbrueckii* subsp. *bulgaricus* DSM 24734, *Bifidobacterium longum* DSM 24736, *Bifidobacterium breve* DSM 24732, *Bifidobacterium infantis* DSM 24737, *Streptococcus thermophilus* DSM 24731 of 1.125 × 10^11^		For 2 months	[49]
No	*n* = 67 patients	*n* = 30 Probiotics *n* = 37 Control	3 months of 1500 mg 3 times/day of *Clostridium butyricum* (CGMCC0313-1) of >1.0 × 10^7^ CFU/g, *Bifidobacterium infantis* (CGMCC0313-2) of 1.0 × 10^6^ CFU/g		NS	[50]
NCT01686698	*n* = 121 initially included, but remained *n* = 36	*n* = 18 Placebo *n* = 18 Probiotics	4.4 g sachet containing 4.5 × 10^11^ 2 times/day (every 12 h) for 12 weeks of *Lactobacillus paracasei* DSM 24733, *Lactobacillus acidophilus* DSM 24735, *Lactobacillus delbrueckii* subsp. *bulgaricus* DSM 24734, *Lactobacillus plantarum* DSM 24730, *Bifidobacterium breve* DSM 24732, *Bifidobacterium longum* DSM 24736, *Bifidobacterium infantis* DSM 24737, *Streptococcus thermophilus* DSM 24731		At baseline, week 6, week 12 (end of treatment), and week 20 (end of study)	[51]
No	*n* = 110 initially included, but remained *n* = 92	*n* = 43 Placebo *n* = 44 *Lactobacillus casei* Shirota	6 months of 65 mL (6.5 × 10^9^ CFU) containing *Lactobacillus casei* Shirota 3 times/day		At baseline, day 0, day 14, 1–3–6 months	[52]
NCT05231772	*n* = 198 initially included, but remained *n* = 40	*n* = 16 Placebo *n* = 24 Probiotics	3 months of 250 mg 2 times/day of *Saccharomyces boulardii*		3 months	[53]

NS—not specified. CFU—colony forming units. BCAAs—branched-chain amino acids. FOS—fructooligosaccharides. ITT—intention-to-treat. LGG—*Lactobacillus* GG.

**Table 2:** All reported precipitating factors encountered in the enlisted patients and the associated ratio based on the number of participants or episodes developed.

Precipitating Factors	Proportion or Number of Patients or Episodes			Reference
Pneumonia Variceal bleed Hyponatremia Constipation	1 1 1 1			[32]
Acute kidney injury (2) **, pneumonia (1) **, anasarca (1) **, lactulose non-adherence, renal insufficiency (without HE) Infections HE Electrolyte abnormalities	1 2 2 1			[34]
Variceal bleed UTI with sepsis SBP Pneumonia with sepsis Constipation Unknown	4 vs. 4 vs. 5 0 vs. 1 vs. 2 4 vs. 3 vs. 7 2 vs. 0 vs. 3 3 vs. 5 vs. 8 5 vs. 9 vs. 12			[44]
Variceal bleed SBP Constipation UTI Pneumonia	2 vs. 3 1 vs. 4 3 vs. 3 1 vs. 2 0 vs. 2			[46]
Variceal bleed Constipation Infections Overall SBP Pneumonia Other infections * Diuretics/renal dysfunction Spontaneous (%)	16 (24.2) vs. 17 (26.6) 37 (56.1) vs. 33 (51.6) - 18 (27.3) vs. 20 (31.3) 14 (21.2) vs. 8 (12.5) 2 (3.0) vs. 6 (9.4) 2 (3.0) vs. 6 (9.4) 4 (6.1) vs. 6 (9.4) 2 (3.0) vs. 1 (1.6)			[48]
Variceal bleed Sepsis Alcoholic hepatitis Superimposed acute vital hepatitis Spontaneous	4 10 2 2 4			[49]
SPB Lower respiratory tract infection UTI Gastroenteritis Occult sepsis Other Ascites Variceal bleed Jaundice HE Hepatorenal syndrome	Day 0 0 vs. 0 2.33 vs. 0 0 vs. 2.27 2.33 vs. 0 0 vs. 0 6.98 vs. 0 2.33 vs. 6.82 0 vs. 0 2.33 vs. 4.55 0 vs. 2.27 0 vs. 2.27	Month 1 0 vs. 0 2.44 vs. 0 0 vs. 0 0 vs. 0 0 vs. 0 2.44 vs. 0 4.86 vs. 5.13 0 vs. 1.27 0 vs. 2.56 0 vs. 0 0 vs. 0	Month 6 0 vs. 0 0 vs. 0 0 vs. 0 0 vs. 0 0 vs. 0 5.72 vs. 3.23 5.71 vs. 6.45 0 vs. 1.52 0 vs. 0 0 vs. 0 0 vs. 0	[52]

SBP—spontaneous bacterial peritonitis, UTI—urinary tract infection, *—superimposed factors, **—number of episodes.

## Data Availability

The datasets used and analyzed during the current study are available from the corresponding author on reasonable request.
